# Copper nanocolloids: a new thrombus molecular imaging approach to ruptured plaque

**DOI:** 10.1186/1532-429X-14-S1-O42

**Published:** 2012-02-01

**Authors:** Dipanjan Pan, Shelton D Caruthers, Angana SenPan, Michael J Scott, Anne H Schmieder, Patrick J Gaffney, Samuel A Wickline, Gregory Lanza

**Affiliations:** 1 Medicine, Washington University Medical School, Saint Louis, MO, USA; 2 Surgery, Saint Thomas', London, UK

## Summary

Molecular imaging of fibrin offers a sensitive way to detect ruptured atherosclerotic plaque with MRI. To date we and others have heavily explored the use of gadolinium and manganese as paramagnetic metals to provide T1 contrast. In this project, we developed the first bivalent copper nanocolloids for MR molecular imaging of thrombus.

## Background

Robust detection of fibrin expressed in the microfissues of ruptured plaque of the carotid artery offers an opportunity to intercede prophylatically in patients at high risk for stroke. Given the abundance of fibrin in microthrombus, we have focused on developing gadolinium-free nanomedicine strategies for paramagnetic imaging of clot, respecting recent concerns regarding the pathological link between the lanthanide and Nephrogenic Systemic Fibrosis in patients with severe renal disease. The objective of this research was to develop and characterize the first molecular imaging (MI) agent for fibrin using copper-based nanocolloids (NanoQ).

## Methods

Nanocolloids incorporating copper (II) complexes were synthesized (Dav=217 nm, ζ=-13mV) and characterized for MI of thrombus. MR properties of NanoQ in suspension were defined at 3.0 T at 25°C. Single slice inversion recovery and multi-echo spin echo sequences were used to calculate the ionic (per metal) and particulate (per particle) relaxivities from serial dilutions. T1-weighted gradient echo imaging of fibrin clots with NanoQ or a control (n=3/group) using fibrin-specific antibodies (NIB5F3) were acquired. In vivo pharmacokinetics and 24 hour biodistribution, and bioelimination of NanoQ were evaluated in rodents.

## Results

The particulate relaxivity of the NanoQ was high, r1=66,000±2200 (s●mmol [NanoQ])-1, while the ionic r1 relaxivitiy (4.3±0.1 (s●mmol [Cu])-1) was similar to Gd-DTPA. The particulate r2 relaxivities were r2=135,000±2900 and ionic r2 relaxivities of 10.4±0.34 (s●mmol [Cu])-1, respectively. NanoQ targeted to fibrin clot phantoms provided strong improvement in contrast-to-noise ratio (CNR) (40x p<0.05), while control clots (non-targeted and targeted no metal) had negligible contrast enhancement. NanoQ given intravenously as a bolus had a long half-life (100 min, n=3) determined by fitting two-compartment bi-exponential model fit (R2>0.99). ICP analysis of tissue copper 24 hours post injection revealed that approximately 90% of the metal was already eliminated from the animal.

## Conclusions

Fibrin-specific NanoQ is a first high T1 relaxivity bivalent copper nanoparticluate MI agent that could afford sensitive noninvasive MR imaging of thrombus associated with ruptured plaques in high risk patients.

## Funding

NIH.

**Figure 1:**
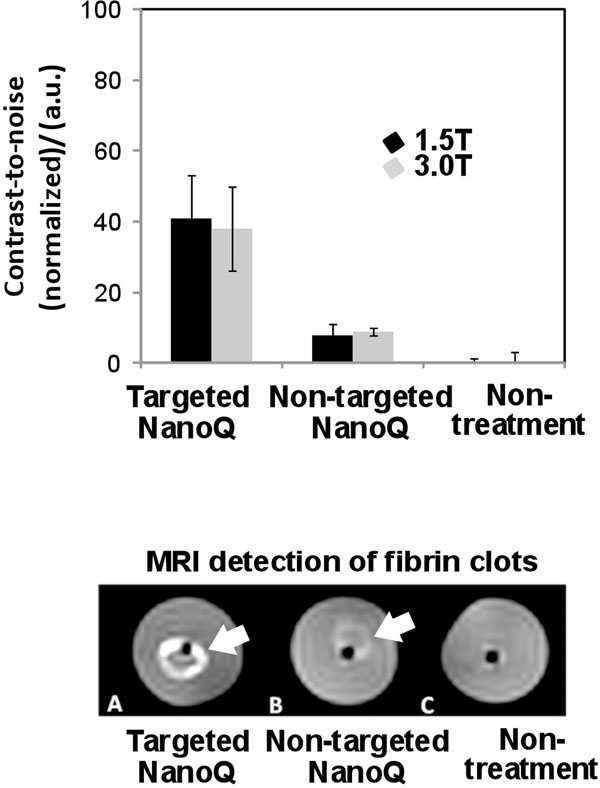
Top: Contrast to Noise ratio (CNR) of fibrin targeted copper nanocolloids (NanoQ) versus nontargeted and control groups at 1.5 and 3T using T1w GRE MR. Bottom: Single slice images of fibrin-rich clots treated with fibrin-targeted NanoQ, nontargeted NanoQ, and control (no treatment) obtained at 3T using a T1w GRE pulse sequence.

